# Gravity and lymphodynamics

**DOI:** 10.14814/phy2.15289

**Published:** 2022-05-19

**Authors:** Thomas Holm‐Weber, Rasmus Eskild Kristensen, Sheyanth Mohanakumar, Vibeke E. Hjortdal

**Affiliations:** ^1^ Department of Cardiothoracic Surgery, Rigshospitalet University of Copenhagen Copenhagen Denmark

**Keywords:** fluid balance, gravity, lymphatics, near‐infrared fluorescence imaging, physiology

## Abstract

The lymphatic system is compromised in different groups of patients. To recognize pathology, we must know what is healthy. We use Near‐Infrared Fluorescence (NIRF) to assess peripheral lymphatic function in humans. We have shown that external factors such as exercise, hyperthermia, and pharmacological mediators influence the function of peripheral lymphatic vessels. In this study, we explored the impact on lymphatic vessels by the ever‐present external factor—*gravity*. We used NIRF imaging to investigate the lymphatic changes to gravity. Gravity was assessed by changing body position from supine to standing. We extracted following lymphatic functional parameters: lymphatic packet propulsion frequency (contractions/min), velocity (cm/s), and pressure (mmHg). Raw data analysis was performed using a custom‐written Labview program. All sequences were analyzed by two observers and interclass correlation scores were calculated. All statistical analysis was performed using RStudio Team (2021). RStudio: Integrated Development Environment for R. RStudio, PBC. Healthy participants (*n* = 17, 11 males, age 28.1 ± 2.6 years) were included. The lymphatic packet propulsion frequency at baseline was 0.5 ± 0.2 contractions/min and rose within 3 min significantly to a maximum of 1.2 ± 0.5 contractions/min during upright posture and remained significantly higher than the baseline lymphatic packet propulsion frequency after lying down again for up to 6 min. The lymph velocity was 1.5 ± 0.4 cm/s at baseline and changed in both directions and without a specific pattern at different points in time during standing. Lymph pressure was significantly higher while standing (mean increase 9 mmHg, CI: 2–15 mmHg). The ICC scores were 89.8% (85.9%–92.7%), 59.3% (46.6%–69.6%) and 89.4% (79.0%–94.8%) in lymphatic packet propulsion frequency (130 observations), velocity (125 observations), and pressure (30 observations), respectively. The lymphatic system responds within few minutes to gravitational changes by increasing lymphatic packet propulsion frequency and pressure.

## Graphical abstract

Gravity affects our Lymphatic system. This study showed the lymphatic response to gravity.

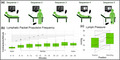

## 1 INTRODUCTION

Interest in the lymphatic system is rising. The lymphatic system is one of the main components in maintaining the fluid balance. But knowledge about how it operates has been limited and foreshadowed by the cardiovascular system. The lack of investigational tools has up until recently been the main reason for this gap. A new investigational tool—the Near‐Infrared Fluorescence (NIRF) imaging—allows us to examine the human lymphatic activity in real‐time (Groenlund et al., 2017; Kelly et al., 2019). Using this tool we have demonstrated how the healthy lymphatic system reacts to external factors such as exercise, hyperthermia, pharmacological mediators—and how the impaired lymphatic system reacts in Fontan patients and breast cancer patients (Alstrup et al., 2021; Belgrado et al., 2016; Groenlund et al., 2017; Marshall et al., 2010; Mohanakumar et al., 2019; Rasmussen et al., 2009, 2010; Sevick‐Muraca et al., 2008; Unno et al., 2008, 2010).

Peripheral edema is a clinical problem for many people and standing increase edema (Junge et al., 2022). Evidently, the edema is a result of the imbalance between filtered fluid from the capillaries and the drainage through the lymphatic system. The higher hydrostatic pressures in both arteries and veins during standing are thought to cause a higher filtration of fluid (Maw et al., 1995). It is, however, unknown exactly how the lymphatic drainage is influenced by standing up and the resulting extra volume load.

We hypothesized that a standing position (and exposure to gravity) and the increased filtration of fluid from the blood capillaries into the interstitial space that follows, would increase lymphatic activity due to an increased workload.

We aimed to investigate the healthy lymphatic system's reaction to change in posture and thereby, the effect of gravity using NIRF imaging in a group of healthy individuals.

## 2 MATERIALS AND METHODS

Near‐infrared Fluorescence Imaging was used to determine the effects of gravity on lymphatic activity.

### 2.1 Near‐Infrared Fluorescence Imaging (NIRF)

Indocyanine Green (ICG; Verdye, Diagnostic Green GmbH, Germany) was excited at wavelengths 750–800 nm and emitted light at 845 nm when returning to the ground state (Miwa, 2010).

The ICG was dissolved in 5 ml of distilled water and then 0.65 ml was extracted and dissolved in 10 ml saline water. The prepared ICG solution concentration was 0.3 mg/ml and each injection (0.1 ml) resulting in a final concentration of 0.03 mg ICG per injection.

The prepared ICG solution was injected intradermally, before the area of interest was excited by a custom‐designed laser; excitation: 750 nm, excitation surface power: >9 mW/cm^2^. The ICG's emission of light was then collected using custom‐designed two complementary metal‐oxide semiconductor cameras recording wavelengths >790 nm (Kaer Labs, Nantes, France).

The frame rate was 1.58 frames per second and determined by two factors: Fluo exposure (catches the fluorescence of ICG) and Bright exposure (produces the canvas of the lymphatic vessels) and was set to 300 and 2 milliseconds, respectively. The laser was set to 100%.

Each participant was injected intradermally in three areas of the right foot: The first and the fourth intermetatarsal space, and behind the medial malleolus. Upon injection, ICG was taken up by the superficial lymphatic vessels and moved centrally. All recordings for data analysis were performed on the right lower leg.

### 2.2 Study design (Figure 1a)

#### 2.2.1 Study population

We enrolled 17 participants—one participant's data had to be excluded because of the poor quality of the footage (Table 1). Inclusion criteria were age between 20 and 35 years and good health. Exclusion criteria were lymphedema, edema or any venous diseases.

**TABLE 1:** Clinical characteristics

	Male		Female	
	Mean	SD	Mean	SD
Age (years)	29	2.7	27	2.6
Height (cm)	185	5.0	168	3.2
Weight (kg)	83	11.2	62	2.8
BMI (kg/m^2^)	24.2	2.2	21.9	1.2
Systolic blood pressure (mmHg)	129	15.1	120	6.3
Diastolic blood pressure (mmHg)	77	13.9	73	6.7
Heart rate (/minute)	65	10.5	68	12.6

Note

Clinical characteristics of the 16 healthy participants in the study (male: *n* = 10).

Based on a standard deviation of 0.1 min^−1^ in mean contraction frequency found in a previous NIRF imaging study by Telinius et al. (2014) inclusion of 16 patients would detect a 12% difference in contraction frequency with a power of 80% and significance level set to *p* < 0.05.

The investigation was conducted in a controlled identical environment for every participant: room temperature was maintained at 23.8 ± 0.9°C; the participants spent 15 min in supine position in a bed for acclimatizing to position and temperature. The total investigation time was approximately 1.5−2 h.

**FIGURE 1:**
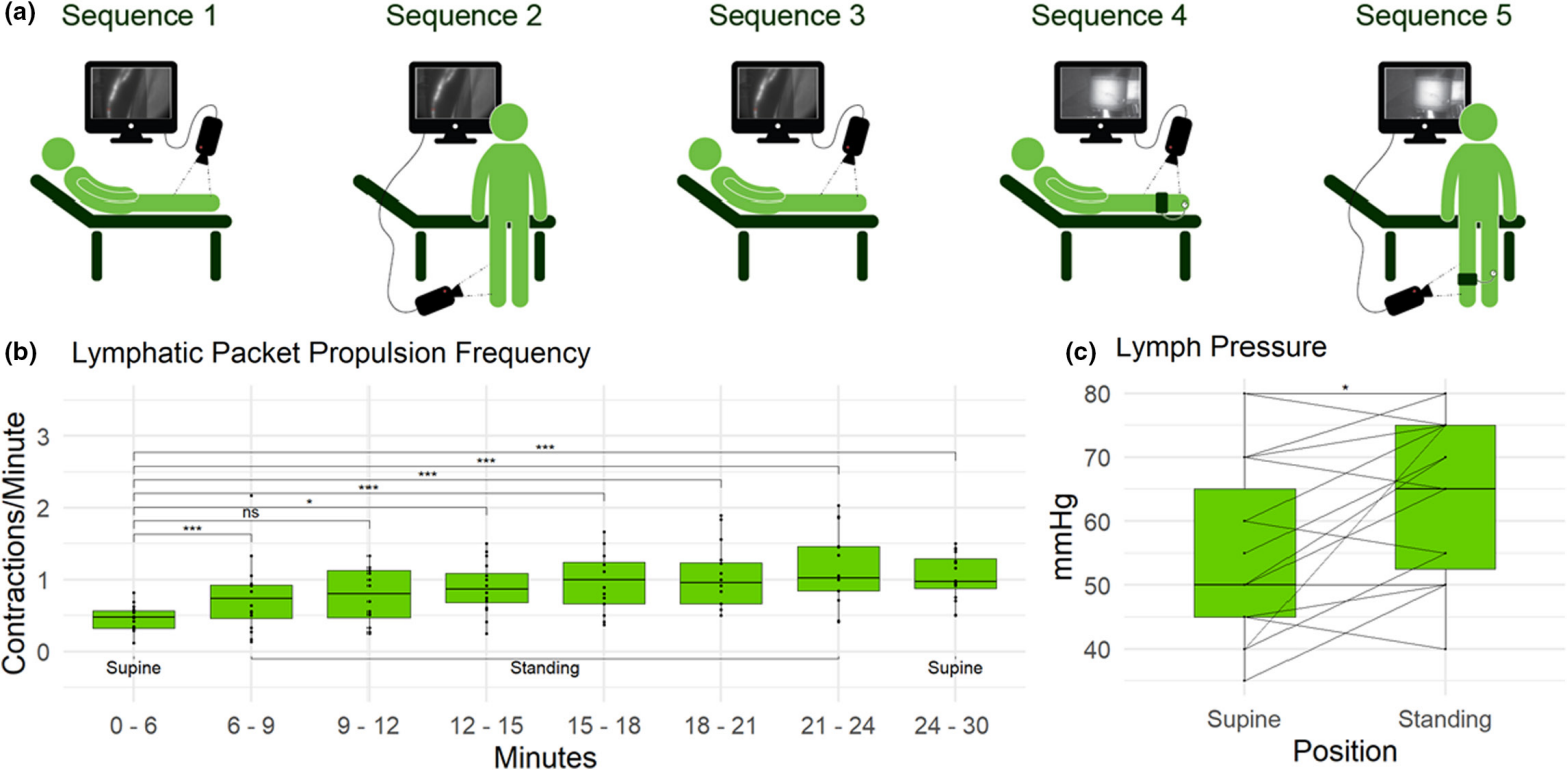
(a) Schematic drawing of the five sequences. Sequences 1–3 recorded lymphatic packet propulsion frequency and lymph velocity. Sequences 4 and 5 recorded lymph pressure. 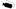: Costum‐built camera to capture the ICG‐emitted light. 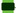: Inflatable cuff used to determine lymph pressure. (b) Changes in lymphatic packet propulsion frequency when changing body position from supine (sequence 1) to standing (sequence 2) and supine again (sequence 3). (c) Lymph pressure while lying down (sequence 4) and standing up (sequence 5)

Upon arrival, each participant's weight, height, standing‐, and supine blood pressure were collected. Next, an injection of ICG was performed and an injection sequence was recorded. Then, the participants had 15 min of rest for allowing the ICG to be distributed throughout all possible vessels. The sequence order was designed to detect changes in the lymphatic packet propulsion frequency, velocity, and pressure. Sequence 1 through 3 examined lymph lymphatic packet propulsion frequency and velocity, whereas, sequence 4 and 5 examined lymph pressure (Figure 1a).

### 2.3 Outcome measures

We assessed lymphatic function by following primary outcomes: lymphatic packet propulsion frequency, velocity, and pressure.

#### 2.3.1 Lymphatic packet propulsion frequency (contractions/min)

We defined the lymphatic packet propulsion frequency as the number of lymphatic packets propelled through a vessel pr. minute.

Regions of interests (ROIs) were plotted on all vessels, the intensity within the ROIs measured, and a differentiated intensity curve was calculated and plotted. A visual contraction was defined as a transient positive deflection followed by a negative.

A contraction was defined as a combination of a measured change in light intensity and a visual confirmation.

The measured change in light intensity was defined as a valley of at least 100 DI/Dt in the differentiated intensity curve and visual confirmation was performed by visually confirming the lymph packet propulsion through the ROI. The number of events was counted and divided by the number of minutes of the sequence.

#### 2.3.2 Lymph velocity (cm/s)

Packet velocity was measured as packets that move continuously through two ROIs with no pauses. Packet velocity was calculated by dividing the packet transit time between the two ROIs with the distance between the ROIs. The valley‐to‐valley time difference on the differentiated intensity curve in the two ROIs was measured. Distances between the ROIs were calculated by using a calibration scale inserted at the beginning of each sequence.

#### 2.3.3 Lymph pressure (mmHg)

Pumping pressure is the maximal pressure that the contractile vessels can develop. We estimated this by inflating a standard sphygmomanometer cuff around the calf, after having manually emptied the lymphatic vessels under and proximally to the cuff. We inflated the cuff to 80 mmHg and reduced the pressure by 10 mmHg every 5 min until lymph passed the cuff. Pumping pressure was measured standing and supine at the same position on the calf.

### 2.4 Data analysis

#### 2.4.1 Sequence analysis

We analyzed the experimental sequences with a custom‐written LabVIEW program (National Instruments, Texas). All sequences were blinded before analysis, the blinding was revealed after completion of all data analysis. All data were analyzed by two raters. The reported lymphatic packet propulsion frequency and velocity were averaged for all visible vessels (average vessels per participant: 2.6 ± 0.8). Time durations for the sequences were: Six minutes (Sequences 1 and 3), 18 min (Sequence 2), and until lymph passed the cuff between 5 and 40 min (Sequences 4 and 5). Sequences 1, 3, 4, and 5 were analyzed in full length. Sequence 2 was divided into six 3‐min parts to determine the specific time of potential changes in lymphatic packet propulsion frequency and velocity.

#### 2.4.2 Statistical analysis

A repeated measures two‐way ANOVA (between and time(within)) was performed to test if standing up changes the lymphatic packet propulsion frequency and velocity.

All three assumptions were met when analyzing lymphatic packet propulsion frequency—the dependent variable was approximately normally distributed and there were no significant outliers. The measurement variance did differ but was revised using Greenhouse‐Geisser correction.

None of the assumptions were met while analyzing lymph velocity.

Lymph pressures were compared using paired *t*‐test. All assumptions were met—there were no significant outliers and the differences were normally distributed.

We used Rstudio two‐way mixed methods Single Score Intraclass correlation (ICC and 95% confidence interval) to analyze for interobserver variance.

### 2.5 Ethical approval

The study was approved by the Central Denmark Region Committees on Health Research Ethics (H‐19087610) and conducted in accordance with the Helsinki declaration. Written informed consent was obtained from all participants.

The study was registered at The Danish Data Protection Agency and conducted in accordance with The General Data Protection Regulation.

## 3 RESULTS

One participant was excluded due to poor imaging quality.

A total of 78 sequences were analyzed and 15 out of the 16 participants included provided all five sequences. One participant only provided three sequences due to technical problems during the lymph pressure sequences.

### 3.1 Lymphatic packet propulsion frequency (contractions/min)

Lymphatic packet propulsion frequency increased significantly within a few minutes and remained high throughout the investigation (Figure 1b).

### 3.2 Lymph velocity (cm/s)

Lymph velocity did not change throughout the investigation (Table 2).

**TABLE 2:** Lymph velocity (cm/s)

	Mean	SD
Sequence 1	1.50	0.37
Sequence 2 Part 1	1.52	0.42
Sequence 2 Part 2	1.37	0.31
Sequence 2 Part 3	1.39	0.29
Sequence 2 Part 4	1.49	0.28
Sequence 2 Part 5	1.70	0.42
Sequence 2 Part 6	1.61	0.49
Sequence 3	1.98	0.75

Notes

Lymph Velocity. Sequence 1: Supine body position, 6 min. Sequence 2: Standing body position, total 18 min, each part 3 min. Sequence 3: Supine after standing, 6 min.

### 3.3 Lymph pressure (mmHg)

Lymph pressure was significantly higher when standing compared to lying down (Figure 1c).

### 3.4 Interobserver variability

The ICC scores was 89.8% (85.9%–92.7%), 59.3% (46.6%–69.6%) and 89.4% (79.0%–94.8%) in lymphatic packet propulsion frequency (130 observations), velocity (125 observations) and pressure (30 observations) respectively.

## 4 DISCUSSION

The lymphatic packet propulsion frequency more than doubled and lymph pressure increased with an average 9 mmHg when moving from supine to standing position within a few minutes. This implies, that the lymphatic system is quickly adapting to gravitational changes.

Lymph velocity was unaffected by gravity, this is in accordance with previous studies examining the effect of external factors on lymph velocity (Groenlund et al., 2017; Kelly et al., 2019; Mohanakumar et al., 2021). The velocity ICC score was the lowest of the three parameters indicating that this parameter is the least reproducible, making it more difficult to document a change.

The response of the lymphatic system to gravitational changes has not been thoroughly investigated before. Rasmussen et al found no significant change in lymphatic activity due to body position in 5 human subjects (Rasmussen et al., 2020). Our increased lymphatic packet propulsion frequency and pressure in a cohort of 16 study subjects complies with a smaller study on mice by Bouta et al. (2018).

Standing position increases capillary pressure and fluid filtration leading to more interstitial fluid, and assumedly results in a bigger workload and shear stress for the lymphatic vessels (Graaff et al., 2003; Maw et al., 1995). Math simulations (Kunert et al., 2015), In‐vitro studies (Telinius et al., 2017), and animal testing (Benoit et al., 1989) have demonstrated that increase in mechanical stretch is a driving factor for initiating lymphatic contractions. Our findings of an increased lymphatic packet propulsion frequency support this theory in healthy adults.

Hyperthermia leads to the increased interstitial fluid formation and has been used to evaluate lymphatic function. Kelly et al. (2019) and Mohanakumar et al. (2021) used NIRF imaging to investigate the effects of hyperthermia on the peripheral lymphatic vessels. Furthermore, Olszewski et al. (1977) investigated the effects of hyperthermia using lymphatic cannulation. All three studies found an increase in lymphatic packet propulsion frequency. During standing and hyperthermia interstitial fluid formation increases, and the findings of hyperthermia studies accord with the findings from this current study.

This study was designed to investigate the effects of gravity on the function of the human lymphatic system—a better understanding of basic lymphatic physiology might lead to a better understanding of pathophysiology followed by better treatment of lymphatic disorders such as lymphedema.

### 4.1 Limitations

NIRF imaging only visualizes the superficial lymphatic vessels and deep vessels might react differently to the gravity. The study investigated the response in young healthy adults, and the response in other age groups might differ.

## 5 CONCLUSION

In this study of the healthy human lymphatic system and its response to gravity, we found a significant increase in lymphatic packet propulsion frequency and lymphatic pressure within few minutes as a response to standing. This knowledge introduces Gravity as a new factor to consider when using NIRF imaging as a tool to evaluate lymphatic functional competence in patients with lymphatic complications.

## AUTHOR CONTRIBUTION

TH, SM, and VH was part of the study design. TH and RK recruited and conducted the NIRF investigations. Data analysis was done by TH and RK. Statistical analysis was performed by TH. All authors contributed to drafting and reviewing the manuscript.

## References

[phy215289-bib-0001] Alstrup, M. , Johannessen, A. L. , Mohanakumar, S. , Offersen, B. V. , & Hjortdal, V. E. (2021). Lymphatic function in the arms of breast cancer patients—A prospective cohort study. Plastic and Reconstructive Surgery ‐ Global Open, 9(8), 3779. 10.1097/GOX.0000000000003779 PMC8386902 34476161

[phy215289-bib-0002] Belgrado, J. P. , Vandermeeren, L. , Vankerckhove, S. , Valsamis, J.‐B. , Malloizel‐Delaunay, J. , Moraine, J.‐J. , & Liebens, F. (2016). Near‐infrared fluorescence lymphatic imaging to reconsider occlusion pressure of superficial lymphatic collectors in upper extremities of healthy volunteers. Lymphatic Research and Biology, 14(2), 70–77. 10.1089/lrb.2015.0040 27167187 PMC4926199

[phy215289-bib-0003] Benoit, J. N. , Zawieja, D. C. , Goodman, A. H. , & Granger, H. J. (1989). Characterization of intact mesenteric lymphatic pump and its responsiveness to acute edemagenic stress. American Journal of Physiology‐Heart and Circulatory Physiology, 257(6), H2059–H2069. 10.1152/AJPHEART.1989.257.6.H2059 2603989

[phy215289-bib-0004] Bouta, E. M. , Blatter, C. , Ruggieri, T. A. , Meijer, E. F. J. , Munn, L. L. , Vakoc, B. J. , & Padera, T. P. (2018). Lymphatic function measurements influenced by contrast agent volume and body position. JCI Insight, 3(2). 10.1172/jci.insight.96591 PMC5821192 29367467

[phy215289-bib-0005] de Graaff, J. C. , Ubbink, D. T. , Lagarde, S. M. , & Jacobs, M. J. H. M. (2003). Postural changes in capillary pressure in the hallux of healthy volunteers. Journal of Applied Physiology, 95(6), 2223–2228. 10.1152/japplphysiol.00210.2003 12871963

[phy215289-bib-0006] Groenlund, J. H. , Telinius, N. , Skov, S. N. , & Hjortdal, V. (2017). A validation study of near‐infrared fluorescence imaging of lymphatic vessels in humans. Lymphatic Research and Biology, 15(3), 227–234. 10.1089/lrb.2016.0061 28749720

[phy215289-bib-0007] Junge, F. , Konschake, W. , Haase, H. , Vollmer, M. , & Jünger, M. (2022). Walking instead of standing: Influence of movement on sensations of discomfort and the volume of the lower legs during standing loads. Vasa ‐ European Journal of Vascular Medicine, 51(2), 78–84. 10.1024/0301-1526/a000990 35142231

[phy215289-bib-0008] Kelly, B. , Mohanakumar, S. , Telinius, N. , Alstrup, M. , & Hjortdal, V. (2019). Function of upper extremity human lymphatics assessed by near‐infrared fluorescence imaging. Lymphatic Research and Biology. Published online September 17, 2019:lrb.2019.0041. 10.1089/lrb.2019.0041 31526221

[phy215289-bib-0009] Kunert, C. , Baish, J. W. , Liao, S. , Padera, T. P. , & Munn, L. L. (2015). Mechanobiological oscillators control lymph flow. Proceedings of the National Academy of Sciences of the United States of America, 112(35), 10938–10943. 10.1073/pnas.1508330112 26283382 PMC4568261

[phy215289-bib-0010] Marshall, M. V. , Rasmussen, J. C. , Tan, I. , Aldrich, M. B. , Adams, K. E. , Wang, X. , Fife, C. E. , Maus, E. A. , Smith, L. A. , & Sevick‐Muraca, E. M. (2010). Near‐infrared fluorescence imaging in humans with indocyanine green: A review and update. The Open Surgical Oncology Journal, 2(1), 12–25. 10.2174/1876504101002010012 22924087 PMC3424734

[phy215289-bib-0011] Maw, G. J. , Mackenzie, I. L. , & Taylor, N. A. S. (1995). Redistribution of body fluids during postural manipulations. Acta Physiologica Scandinavica, 155(2), 157–163. 10.1111/j.1748-1716.1995.tb09960.x 8669288

[phy215289-bib-0012] Miwa, M. (2010). The principle of ICG fluorescence method (vol. 2). Retrieved from http://jp.hamama

[phy215289-bib-0013] Mohanakumar, S. , Kelly, B. , Turquetto, A. L. R. , Alstrup, M. , Amato, L. P. , Barnabe, M. S. R. , Silveira, J. B. D. , Amaral, F. , Manso, P. H. , Jatene, M. B. , & Hjortdal, V. E. (2021). Functional lymphatic reserve capacity is depressed in patients with a Fontan circulation. Physiological Reports, 9(11). 10.14814/PHY2.14862 PMC8165731 34057301

[phy215289-bib-0014] Mohanakumar, S. , Telinius, N. , Kelly, B. , Lauridsen, H. , Boedtkjer, D. , Pedersen, M. , de Leval, M. , & Hjortdal, V. (2019). Morphology and function of the lymphatic vasculature in patients with a fontan circulation. Circulation: Cardiovascular Imaging, 12(4), e008074. 10.1161/CIRCIMAGING.118.008074 30943769

[phy215289-bib-0015] Olszewski, W. , Engeset, A. , Icger, P. M. , Sokolowski, J. , & Theodorsen, L. (1977). Flow and composition of leg lymph in normal men during venous stasis, muscular activity and local hyperthermia. Acta Physiologica Scandinavica, 99(2), 149–155. 10.1111/j.1748-1716.1977.tb10365.x 842371

[phy215289-bib-0016] Rasmussen, J. C. , Kwon, S. , Pinal, A. , Bareis, A. , Velasquez, F. C. , Janssen, C. F. , Morrow, J. R. , Fife, C. E. , Karni, R. J. , & Sevick‐Muraca, E. M. (2020). Assessing lymphatic route of CSF outflow and peripheral lymphatic contractile activity during head‐down tilt using near‐infrared fluorescence imaging. Physiological Reports, 8(4). 10.14814/phy2.14375 PMC7058174 32097544

[phy215289-bib-0017] Rasmussen, J. C. , Tan, I.‐C. , Marshall, M. V. , Adams, K. E. , Kwon, S. , Fife, C. E. , Maus, E. A. , Smith, L. A. , Covington, K. R. , & Sevick‐Muraca, E. M. (2010). Human lymphatic architecture and dynamic transport imaged using near‐infrared fluorescence Translational Oncology, 3, 362–372. 10.1593/tlo.10190 21151475 PMC3000461

[phy215289-bib-0018] Rasmussen, J. C. , Tan, I. C. , Marshall, M. V. , Fife, C. E. , & Sevick‐Muraca, E. M. (2009). Lymphatic imaging in humans with near‐infrared fluorescence. Current Opinion in Biotechnology, 20(1), 74–82. 10.1016/j.copbio.2009.01.009 19233639 PMC2692490

[phy215289-bib-0019] Sevick‐Muraca, E. M. , Sharma, R. , Rasmussen, J. C. , Marshall, M. V. , Wendt, J. A. , Pham, H. Q. , Bonefas, E. , Houston, J. P. , Sampath, L. , Adams, K. E. , Blanchard, D. K. , Fisher, R. E. , Chiang, S. B. , Elledge, R. , & Mawad, M. E. (2008). Imaging of lymph flow in breast cancer patients after microdose administration of a near‐infrared fluorophore: Feasibility study. Radiology, 246(3), 734–741. 10.1148/radiol.2463070962 18223125 PMC3166516

[phy215289-bib-0020] Telinius, N. , Drewsen, N. , Pilegaard, H. , Kold‐Petersen, H. , de Leval, M. , Aalkjaer, C. , Hjortdal, V. , & Boedtkjer, D. B. (2010). Human thoracic duct in vitro: Diameter‐tension properties, spontaneous and evoked contractile activity. American Journal of Physiology‐Heart and Circulatory Physiology, 299(3). 10.1152/ajpheart.01089.2009 20511415

[phy215289-bib-0021] Telinius, N. , Majgaard, J. , Mohanakumar, S. , Pahle, E. , Nielsen, J. , Hjortdal, V. , Aalkjær, C. , & Boedtkjer, D. B. (2017). Spontaneous and evoked contractility of human intestinal lymphatic vessels. Lymphatic Research and Biology, 15(1), 17–22. 10.1089/lrb.2016.0039 28277905

[phy215289-bib-0022] Telinius, N. , Mohanakumar, S. , Majgaard, J. , Kim, S. , Pilegaard, H. , Pahle, E. , Nielsen, J. , de Leval, M. , Aalkjaer, C. , Hjortdal, V. , Boedtkjer, D. B. , & Boedtkjer, D. B. (2014). Human lymphatic vessel contractile activity is inhibited in vitro but not in vivo by the calcium channel blocker nifedipine. The Journal of Physiology, 592, 4697–4714. 10.1113/jphysiol.2014.276683 25172950 PMC4253471

[phy215289-bib-0023] Unno, N. , Nishiyama, M. , Suzuki, M. , Tanaka, H. , Yamamoto, N. , Sagara, D. , Mano, Y. , & Konno, H. (2010). A novel method of measuring human lymphatic pumping using indocyanine green fluorescence lymphography. Journal of Vascular Surgery, 52(4), 946–952. 10.1016/j.jvs.2010.04.067 20619581

[phy215289-bib-0024] Unno, N. , Nishiyama, M. , Suzuki, M. , Yamamoto, N. , Inuzuka, K. , Sagara, D. , Tanaka, H. , & Konno, H. (2008). Quantitative lymph imaging for assessment of lymph function using indocyanine green fluorescence lymphography. European Journal of Vascular and Endovascular Surgery, 36(2), 230–236. 10.1016/j.ejvs.2008.04.013 18534875

